# Implementation of strategies to increase adolescents’ access to fruit and vegetables at school: process evaluation findings from the Boost study

**DOI:** 10.1186/s12889-015-1399-9

**Published:** 2015-02-06

**Authors:** Anne Kristine Aarestrup, Thea Suldrup Jørgensen, Sanne Ellegaard Jørgensen, Deanna M Hoelscher, Pernille Due, Rikke Krølner

**Affiliations:** Centre for Intervention Research in Health Promotion and Disease Prevention, National Institute of Public Health, University of Southern Denmark, Øster Farimagsgade 5A 2nd floor, 1353 Copenhagen K, Denmark; Michael & Susan Dell Center for Healthy Living, The University of Texas School of Public Health, Austin Regional Campus, 1616 Guadalupe, Suite 6.300, Austin, Texas 78701 USA

**Keywords:** Process evaluation, Implementation, School, Availability, Accessibility, Fruit and vegetables, Adolescents

## Abstract

**Background:**

Access to fruit and vegetables (FV) is associated with adolescents’ FV consumption. However, little is known about implementation of strategies to increase access to FV at schools. We examined the implementation of two environmental components designed to increase access to FV at Danish schools.

**Methods:**

We used data from 20 intervention schools involved in the school-based multicomponent Boost trial targeting 13-year-olds’ FV consumption. The environmental components at school included daily provision of free FV and promotion of a pleasant eating environment.

Questionnaire data was collected by the end of the nine-month intervention period among 1,121 pupils (95%), from all school principals (n = 20) and half way through the intervention period and by the end of the intervention among 114 teachers (44%).

The implementation of the components was examined descriptively using the following process evaluation measures; fidelity, dose delivered, dose received and reach. Schools with stable high implementation levels over time were characterised by context, intervention appreciation and implementation of other components.

**Results:**

For all process evaluation measures, the level of implementation varied by schools, classes and over time. Dose received: 45% of pupils (school range: 13-72%, class range: 7-77%) ate the provided FV daily; 68% of pupils (school range: 40-93%, class range: 24-100%) reported that time was allocated to eating FV in class. Reach: The intake of FV provided did not differ by SEP nor gender, but more girls and low SEP pupils enjoyed eating FV together. Dose delivered: The proportion of teachers offering FV at a daily basis decreased over time, while the proportion of teachers cutting up FV increased over time. Schools in which high proportions of teachers offered FV daily throughout the intervention period were characterized by being: small; having a low proportion of low SEP pupils; having a school food policy; high teacher- and pupil intervention appreciation; having fewer teachers who cut up FV; and having high implementation of educational components.

**Conclusions:**

The appliance of different approaches and levels of analyses to describe data provided comprehension and knowledge of the implementation process. This knowledge is crucial for the interpretation of intervention effect.

**Trial registration:**

Current Controlled Trials ISRCTN11666034

## Background

Inadequate fruit and vegetable (FV) consumption is associated with the development of obesity, cardiovascular disease and cancer [1,2]. Children and adolescents in Western countries do not reach the recommended level of intake of fruit and vegetables [3,4] and a number of school-based interventions have been conducted mainly among children aged 12 or less. As intake of FV decreases from age 11 to 15 [5] adolescents are an important target group for interventions, although challenging due to the significant developmental changes they undergo in this life period, psychologically, physically and socially [6]. High availability (i.e. presence of FV at school) and accessibility (i.e. cutting up FV, time to eat FV) of FV are associated with high consumption of FV among adolescents, and it is recommended that interventions target these determinants [7]. These challenges prompted the development of the multicomponent Boost intervention aimed at increasing FV intake in 13-year-old Danish adolescents.

The level of implementation of an intervention is likely to affect intervention effectiveness [8]. A thorough process evaluation of the implementation level is therefore important in order to interpret intervention outcomes correctly [8,9].

Few studies have reported implementation levels of strategies to increase availability and accessibility of FV through FV programmes at school. Potter et al. [10] addressed availability and accessibility of FV through a multicomponent intervention providing free FV for *all pupils* at 25 schools in Mississippi and reported on implementation (e.g. variety of FV served), on who was involved in the implementation (FV suppliers, principals, teachers) and on how it was received (pupils satisfaction) [10]. It was concluded that the implementation was successful and that the programme was well received by the participants. In a parent-financed FV subscription programme among year 6 pupils (11 years), Bere et al. [11] reported that 4 of the 9 Norwegian intervention schools participated in the FV programme while 42 (22%) and 28 (15%) of the 190 pupils enrolled subscribed at first and at second follow-up [11]. In a FV programme targeting 10-13-year-olds in three countries [12], Wind et al. (2008) found that all Dutch pupils received FV provided. While in Norway and Spain, few children changed their existing subscription status as a response to the intervention of a school fruit programme [12]. Nathan et al. [13] found that less than half of Australian schools met the public recommendation of having a FV break to grant 80% of the children (5–12 years) eat FV brought from home. Small schools, rural schools and schools from lower socioeconomic areas were more likely to meet the recommendation [13]. Reviewing existing studies reveal that implementation levels have only been reported in few studies using different approaches and levels of description of results.

Data collection at multiple time points is important as studies indicate that implementation may deteriorate over time [8,14-19]. This implies that assessment of the implementation level at the beginning of the intervention period may overestimate the overall implementation level [8]. Furthermore, temporal changes in implementation may give important insights into the working mechanisms of the intervention. Studies assessing the implementation level of environmental strategies to increase adolescents’ FV intake at multiple time points are lacking.

Boys and adolescents with low socio economic position (SEP) eat less FV compared to girls and high SEP adolescents [20]. To our knowledge few studies have examined how well interventions reach these vulnerable subgroups [9]. In the qualitative process evaluation of the Boost study, the social aspects of the FV programme i.e. eating FV together in the classroom was more valued by girls participating in focus groups interviews compared to boys. It is unknown whether these qualitative findings may be generalised to the entire Boost population [21]. Studies on differential intervention effectiveness have found that school-based interventions appear to be more effectively improving girls’ dietary behaviour compared to that of boys [22]. Providing free FV appears to be an effective strategy when aiming at reaching all pupils irrespective of their socioeconomic background [23]. Differential effectiveness of interventions may be due to differential implementation of interventions. This issue has not been investigated in previous studies.

Implementation of school-based interventions relies to a great extent on teachers. Knowledge on characteristics of teachers and schools that achieve a high implementation level may benefit the development of more feasible interventions. The implementation level is likely to be affected by organisational support; teachers’ attitude towards the intervention; characteristics of the intervention e.g. interventions which consist of many components may be more difficult to implement due to requirements concerning skills and coordination; and participants’ responsiveness [24,25] e.g. teachers do not implement certain components of health promotion interventions if pupils are not responding or interested [26].

In summary, there is a lack of knowledge about the implementation level of strategies to increase availability and accessibility of FV to adolescents at schools, the reach of strategies in different subgroups of pupils; changes in implementation level over time, and characteristics of schools with high implementation levels.

The aim of this study was to conduct a comprehensive assessment of the implementation of two components designed to increase accessibility and availability of FV to adolescents in a school-based intervention. The study investigated the following four research questions:

1. Do implementation levels diminish over time for all process evaluation measures and at all schools?
2. Do the implementation levels vary by school and by class within schools?
3. Does daily provision of free FV at schools and promotion of social aspects of eating FV together in the classroom reach boys and girls and high and low SEP pupils equally?
4. What characterises schools with a stable high implementation level of intervention components during the entire intervention period?

## Methods

### The Boost study

The aim of the Boost study was to increase FV consumption among 13-year-olds (school year 7) by improving access to FV at school, in families and local communities. The development of the intervention, implementation and evaluation was guided by the Intervention Mapping™ protocol [27]. The Boost study was implemented for nine months in the school year of 2010/2011. The multicomponent intervention consisted of 1) school components: *Daily provision of free FV*, *A pleasant eating environment*, *Classroom curricular activities*, 2) a family component: *Parental newsletters*, *Guided child*–*parent activities and a parent school meeting*, and 3) a local community component: *Information sheets to managers and coaches of sports*- *and youth clubs* [28]. To facilitate implementation, intervention schools were asked to appoint two teachers as local Boost coordinators.

This paper focuses on the two components in the school environment targeting increased availability (*Daily provision of free FV*) and accessibility (*A pleasant eating environment*).

*Daily provision of free FV*: year 7 pupils at intervention schools were provided one piece of fruit or vegetable for free each school day. One serving of FV is for example one apple or one big piece of cucumber. The definition of a serving fits the Danish recommendations which relate to pieces of fruit or vegetables. The free provision should ensure equal access to FV for all adolescents irrespective of socioeconomic background. Local FV suppliers were assigned to deliver varied and high quality FV at schools in the morning twice a week (covering all school days) during the intervention period.

*A pleasant eating environment*: teachers were encouraged to implement a daily FV break during a class lesson or a break where pupils could eat the provided FV together. Each school class was provided a class kit including tools for cutting up FV. The teachers were encouraged to designate FV hosts among the pupils of each class to be responsible for bringing the FV to the classroom, cutting it up in appealing snacks if the fruit was not already easily accessible as for instance for grapes or bananas, serving it to their classmates and cleaning afterwards. Furthermore, teachers were encouraged to eat FV with the pupils [28]. The intention was to increase accessibility of FV and create a pleasant FV eating environment for the pupils.

Two teachers from each intervention school, preferably the local coordinators, were invited to a *one*-*day pre*-*intervention workshop* to prepare them for implementation of the Boost intervention. These teachers were to bring information back to their school and motivate the rest of the year 7 teacher team. We distributed implementation manuals to schools and FV suppliers including: a manual for suppliers on delivery of FV, a teacher manual on conducting school FV breaks, posters with tips for cutting up FV snacks, and hygiene guidelines to be posted in the classroom.

The effectiveness of the Boost intervention was tested in a cluster-randomised controlled study design with 20 intervention- and 20 control schools randomly selected from a random sample of ten Danish municipalities. The qualitative and quantitative evaluation of the implementation of the intervention components were guided by a systematic process evaluation protocol developed specifically for the Boost study [29]. The protocol built on conceptual frameworks and process evaluation concepts defined by Baranowski et al. (2000), Steckler et al. (2002) and Saunders et al. (2005) [9,30,31]. The findings from the qualitative studies have been reported elsewhere [21,32].

### Study sample

The study sample included 20 intervention schools with a total of 55 classes. A total of 1,121 pupils (95% of enrolled pupils at intervention schools) answered web-based questionnaires at baseline (August 2010) and among them 1,060 (91%) completed the follow-up questionnaire by the end of the intervention (May 2011). More boys than girls were lost to follow-up, (borderline significant; P = 0.0532) while there were no significant differences in attrition by SEP or pupils’ daily FV intake at baseline. We included process evaluation questions in a computer tailoring module. Pupils were asked to complete the module three times (September, December and May) during the school year as part of the curricular activities [28]. Computer tailoring was thus used as an additional tool to collect process data in the Boost study to get several measurements throughout the intervention period. The module was completed by 915 pupils (86% out of those with a follow-up questionnaire). Of these respondents, 631 pupils (69%) responded once, 223 pupils (24%) responded twice and 59 pupils (6%) responded all three times. The pupils who completed the module once did not differ from the pupils completing the module two or three times in terms of gender, SEP or daily FV intake at baseline.

At all intervention schools, teachers completed web-based questionnaires midway of the intervention (January/February 2011) and again by the end of the intervention period (May 2011). The midway survey was completed by 114 (44%) teachers and also 114 teachers (44%) completed the survey at the end of intervention. The analyses of changes of implementation level over time only include data from teachers who had completed both the midway and the follow-up surveys (n = 69). This reduced the number of teachers and schools included in some analyses. All school principals completed a web based questionnaire on structural, physical and social characteristics of their school at baseline (n = 20). Completed parent questionnaires were received for 423 students (40%) at follow-up.

### Measures

#### Process evaluation concepts

The implementation was assessed by four key process evaluation concepts which we operationalized as follows [9,29]:

- *Fidelity*: extent to which the two intervention components were delivered by FV supplier and teachers according to the manual for suppliers on delivery of FV and the teacher manual on conducting school FV breaks.
- *Dose delivered*: the proportion of the two components which was delivered by FV suppliers and teachers to the pupils.
- *Dose received*: the extent to which pupils received the two components.
- *Reach*: proportion of pupils in different subgroups (gender and SEP) who received the intervention components.

In Table 1 the data for the two intervention components is grouped according to the covered process evaluation concepts and the operationalization of the concepts are specified.

**Table 1:** **Operationalisation of process evaluation concepts**

**Process evaluation concept**	**Teachers**		**Pupils**
	**Midway survey**	**Follow-up survey**	**Follow-up survey**
**Component 1: Daily provision of free FV**			
***Fidelity to the intervention by FV suppliers***			
*Freshness of FV*	The FV were always fresh at delivery: *fully agree*/*agree* versus *neither agree nor disagree*/*disagree*/*fully disagree*/*do not know* ^#^	The FV were fresh when delivered: *always*/*most of the times* versus *sometimes*/*seldom*/*never*/do not know*	How often the FV were fresh e.g. not too old: *every day*/*most days* versus *some days*/*seldom*/*never*
*Appearance of FV*	The FV always appeared nice and appetizing: *fully agree*/*agree* versus *neither agree nor disagree*/*disagree*/*fully disagree*/*do not know* ^#^	The FV appeared nice and appetizing: *always*/*most of the times* versus *sometimes*/*seldom*/*never*/do not know*	How often the FV appeared nice and appetizing: *every day*/*most days* versus *some days*/*seldom*/*never*
*Variety of FV*		Boost gave pupils the possibility to taste many kinds of fruit (vegetables): *fully agree*/*agree* versus *neither agree nor disagree*/*disagree*/*fully disagree*/*do not know*	Did pupils taste many different fruits (vegetables): *fully agree*/*agree* versus *neither agree nor disagree*/*disagree*/*fully disagree*
***Dose delivered by FV suppliers and teachers***			
*From FV supplier to schools*	How stable the FV delivery at school had been each month since the beginning of the intervention period: *twice a week* versus *once a week*/*once every second week*/*once a month*/*no delivery*/*do not know*"	How stable the FV delivery to school had been each month since the midway survey?: *twice a week* versus *once a week*/*once every second week*/*once a month*/*no delivery*/*do not know*"	
*From teachers to pupils*	How often they offered pupils the delivered Boost FV: *5 days a week* versus *less than 5 days a week* ^¤^	How often they offered pupils the delivered Boost FV: 5 days a week versus less than 5 days a week^¤^	
***Dose received by pupils***			
*Quantity*	Was there *enough* FV for all year 7 pupils to get one piece per day: *every day* versus *most days*/*few days*/*never* ^¤^	Was there *enough* FV for all year 7 pupils to get one piece a day: *every day* versus *most days*/*few days*/*never* ^¤^	How often was there enough FV for all pupils: *every day* versus *most days*/*seldom*/*never*.
*Frequency*			How often did pupils eat the Boost FV: *every day* versus *most days*/*seldom*/*never*.
			Computer tailoring data: Did pupils eat the Boost FV last time the teachers offered it at school: *yes* versus *no*
*Reach*			
			How often did pupils eat the Boost FV: *every day* versus *most days*/*seldom*/*never*(dose received) stratified by gender and SEP.
**Component 2: A pleasant eating environment**			
***Fidelity and dose delivered by teachers***			
	How often were the Boost FV cut up in their lessons: e*very time*/*most times* versus *sometimes*/*seldom*/*never*	How often were the Boost FV cut up in their lessons: e*very time*/*most times* versus *sometimes*/*seldom*/*never*	How often were the Boost FV cut up: *every day*/*most days* versus *some days*/*seldom*/*never*
***Dose received by pupils***			
			Had time been allocated to eat Boost FV in class: *yes* versus *no*
		Did the pupils have a good time when eating Boost FV together: *fully agree*/*agree* versus *neither agree nor disagree*/*disagree*/*fully disagree*	Did pupils have a good time while eating FV together in the classroom: *fully agree*/*agree* versus *neither agree nor disagree*/*disagree*/*fully disagree*
***Reach***			
			Did the pupils have a good time while eating FV together in the classroom: *fully agree*/*agree* versus *neither agree nor disagree*/*disagree*/*fully disagree* stratified by gender and SEP

FV: fruit and vegetables.

SEP: socioeconomic position.

^#^: Measured among teachers who distributed FV in their classes.

*: Classroom teachers and Boost coordinators.

": Boost coordinators.

^¤^: Classroom teachers.

Mistakenly information on variety of FV was unfortunately not collected in the midway survey.

### SEP

Based on pupil baseline data (replaced by follow-up data if missing) on parents’ job title and workplace parents were coded into occupational social classes I-V or group VI economically inactive parents according to standardized coding principles [33,34]. Family social class was based on the highest ranking parent. We divided the family social class variable into high SEP (I - II), medium SEP (III-IV), and low SEP (V and VI).

### Assessment of implementation levels at the individual, class- and school level

Individual level: We calculated the proportions of pupils and teachers participating in the survey who had reported sufficient implementation according to the variables presented in Table 1. The school- and class range were reported to describe variations in implementation level in the various classes and schools.

School-and class level: Teacher data on implementation could not be linked to specific school classes as teachers within the same school were responsible for teaching a number of year 7 classes. Instead we aggregated the teacher data for fidelity, dose delivered, and dose received to the school level in order to assess the implementation level at each school. The pupil data could be aggregated to both class- and school level. We calculated the number of schools and classes who had implemented the two components to a high level. High implementation level was defined pragmatically as 80% or more, as no schools had implemented 100% of the components. We hypothesized that an 80% implementation level would yield greater intervention benefits and outcomes compared to an implementation level below.

### School typology of implementation over time

Based on teacher data collected midway and by the end of the intervention period we constructed a school typology regarding changes in implementation levels over time, consisting of four school types:

- *Schools with stable high implementation level* (*SH*): The implementation level was high (≥80%) at both times of assessments (the ideal situation).
- *Schools with an increase in implementation level* (*I*): The intervention was implemented to a higher extent by the end of intervention period compared to the level at the beginning of the intervention (numeric increase).
- *Schools with a decrease in implementation level* (*D*): The intervention was implemented to a lower extent by the end of the intervention period compared to the beginning of the intervention (numeric decrease).
- *Schools with a stable low implementation level* (*SL*): The implementation level was low (<80%) at both times of assessments.

### Context and appreciation

We used the process evaluation concepts *context* and *appreciation* to characterise the schools with a stable high implementation level throughout the intervention period.

*Context* includes school level factors such as policies, economy, organisational climate, access to canteen, student composition which may influence programme implementation [9]. *Appreciation* describes how FV suppliers, teachers and pupils experience and appreciate the intervention [29].

Based on school principal baseline data, we included a number of contextual factors hypothesized to influence the development in implementation over time:

- *Socioeconomic pupil composition*: Based on the percentage of pupils from families with few economic resources schools were dichotomised into high school SEP (‘maximum 9 % or less’) versus low (‘10% or more’).
- *Having a free or parent financed* FV scheme in year 6 classes during the previous school year prior to the intervention: yes/no.
- *Having a school food policy*: yes/no. Our definition of a school food policy is the presence of written material declaring what kind of food the school allows at the school. The policy could both be concerned with what they sell at the school and what/whether the pupils are allowed to bring certain foods to the school.
- *School size*: The average number of pupils in year 7 at each school (n = 53) was used as a proxy for school size and dichotomized into large schools (at least 53 pupils) versus small schools (less than 53 pupils).
- *Pupils*’ *appreciation of the Boost intervention*: Based on pupil follow-up data on how much they had appreciated participating in the Boost project, pupils were dichotomized into high pupil appreciation (really liked /liked to participate) versus low (neither liked nor disliked/did not like/did not like it at all/do not know the Boost project).
- *Teachers*’ *appreciation of the FV provision*: *Based on teachers*’ *agreement to the statement*: ”The Boost FV scheme worked well” (follow-up data), teachers were dichotomised into high teacher appreciation (totally agree/agree) versus low (neither agree nor disagree/disagree/totally disagree).

Furthermore we characterized schools with a stable high implementation level according to Implementation level of other intervention components:

- *Implementation level of curricular component*: based on the number of curricular activities aiming to increase the pupils’ FV knowledge and skills, teacher reported they had conducted during the intervention year (teacher follow-up data), schools were dichotomised into schools with high implementation levels (at least 5 curricular activities out of 12) and low implementation levels (less than 5 curricular activities out of 12).
- *Implementation level of parental component*: based on Boost coordinators’ information about the number of parental newsletters they had uploaded during the implementation period, schools were dichotomised into high implementation level (4–6 newsletters) versus low (0–3 newsletters). At 6 schools information from Boost coordinators was missing and replaced by parent follow-up data concerning dose received of newsletters.

### Data analysis

We examined differential reach of the intervention components by chi-square tests. Test for trend were performed to test for social gradients in intervention reach. Fisher’s exact tests were performed to compare schools with stable high implementation levels with schools with low or unstable implementation. A 5% significance level was used. Data were analysed using SAS version 9.3.

### Ethical considerations

The Boost study adheres to all Danish ethical standards and has been approved by the Danish data protection agency (J.nr. 2010-54-0974). Parents who did not want their children to participate in the evaluation of the Boost study were able to indicate this in the parental baseline questionnaire and in follow-up questionnaires (passive consent). Responses were treated anonymously and confidentially.

## Results

### Implementation of ’Daily provision of free FV’

Table 2 summarizes the assessment of implementation of *Daily provision of free FV* and Table 3 the changes in schools’ implementation levels (teacher data) of each process measure throughout the implementation period. The results are in the following grouped according to implementation chains: 1) from FV supplier to school, 2) from school to class, 3) from class to pupils.

**Table 2:** **Implementation of** ***Daily provision of free FV*** **: assessed at the individual-, school- and class level**

**Process evaluation concept**	**Source and timing of data collection**						
	**Teacher midway survey**		**Teacher follow-up survey**		**Pupils follow-up survey**		
	**Individual level: average implemen-tation level (A)**	**School level: no of schools ≥80% implementation/all responding schools**	**Individual level: average implementation level (A)**	**School level: no of schools ≥80% implementation/all responding schools**	**Individual level: average implementation level**	**School level: No of schools ≥80% implementation/all responding schools**	**Class level: no of classes ≥80% implementation/all responding classes**
**Fidelity**							
FV are always fresh at delivery	79% (0-100%)	10/17	94% (0-100%)	15/17	68% (A: 46-83%, B:17-100% )	2/20	13/55
FV always appear nice and appetizing	76% (0-100%)	11/17	82% (0-100%)	11/17	64% (A: 42-85%, B: 13-100%)	2/20	11/55
Pupils have been able to taste many different kinds of fruit			87% (0-100%)	14/18	73% (A: 57-93%, B: 35-100% )	4/20	13/55
Pupils have been able to taste many different kinds of vegetables			64% (0-100%)	7/18	50% (A: 27-86%, B: 10-100%)	1/20	2/55
**Dose delivered**							
FV was delivered twice a week							
September	45% (0-100%)	7/17					
October	68% (0-100%)	11/17					
November	82% (0-100%)	14/17					
December	86% (0-100%)	15/17					
January	86% (0-100%)	15/17					
February			89% (0-100%)	13/14			
March			83% (0-100%)	12/14			
April			89% (0-100%)	13/14			
May			89% (0-100%)	13/14			
June			67% (0-100%)	9/14			
The pupils were offered Boost FV 5 days a week	85% (0-100%)	12/17	64% (0-100%)	6/17			
**Dose received**							
Enough FV for every pupil to get one piece a day	58% (0-100%)	5/17	55% (0-100%)	6/17	44% (A: 23-63%, B: 10-80%)	0/20	2/55
Pupils ate FV last time they were in school (CT data)							
1st period					89%s		
2nd period					87%		
3rd period					89%		
Pupils ate Boost FV daily					45% (A: 13-72%, B: 7-77%)	0/20	0/55
**Reach of intervention among subgroups**							
How often have the pupils eaten the Boost FV							
High SEP					46%		
Medium SEP					45%		
Low SEP					42% (P = 0.6941)		
Girls					42%		
Boys					48% (P = 0.0543)		

A: School range: minimum and maximum percentage of teachers/pupils identified across schools.

B: Class range: minimum and maximum percentage of pupils identified across schools.

CT: Computer tailoring.

FV: fruit and vegetables.

SEP: socioeconomic position.

**Table 3:** **Stability in teachers’ implementation over time at each school**

**School**	**Fidelity: fresh FV midway - 1. Fu**	**Fidelity: FV appearance midway - 1. fu**	**Fidelity/dose delivered: FV cut up midway - 1. fu**	**Dose delivered: FV everyday midway - 1. fu**	**Dose delivered: stable delivery**		**Dose received: enough FV midway - 1. fu**
					**September -January**	**January - June**	
**1**	SH	SH	I	D	SH	SH	SL
**2**	SH	D	SH	D	SL	.	D
**3**	I	SL	SL	D	.	.	D
**4**	I	SH	I	SL	SH	SH	SH
**5**	SH	SH	I	SH	SH	SH	SL
**6**	I	I	SL	SH	SH	SH	I
**7**	SH	D	D	SH	I	.	SL
**8**	.	.	SL	.	I	.	.
**9**	.	.	.	.	I	.	.
**10**	.	.	SL	.	.	.	.
**11**	SH	SH	SL	SH	SL	SL	SH
**12**	SL	SL	SH	SL	I	.	SL
**13**	SH	SH	SL	SL	SH	SH	SH
**14**	SH	SH	SH	SH	SH	SH	SL
**15**	SL	SL	SL	D	I	D	SL
**16**	SH	SH	D	D	SH	D	I
**17**	I	I	I	D	I	D	SL
**18**	I	I	I	SH	I	D	SL
**19**	SH	SH	SH	SL	.	.	SH
**20**	SH	SH	SH	D	I	SH	SL
ᅟ	ᅟ	ᅟ	ᅟ	ᅟ	ᅟ	ᅟ	ᅟ
**I: Increase**	5	3	5	0	8	0	2
**SH: Stable high**	10	9	5	6	7	7	4
**SL: Stable low**	2	3	7	4	2	1	9
**D: Decrease**	0	2	2	7	0	4	2

.: no school information available.

From FV supplier to schools:

*Dose delivered by FV suppliers*: The proportion of classroom teachers reporting FV suppliers to deliver FV twice a week almost doubled from September to January (from 45% to 86%) and decreased from February to June (from 89% to 67%) (Table 2).

FV suppliers’ fidelity:

Freshness and appearance of the delivered FV

Individual level: On average, a high proportion (>75%) of teachers reported the FV delivered to be fresh and appealing and this proportion increased from the beginning to the end of the intervention period. We found large variations between schools in the proportion of teachers (from 0% of teachers to 100%) reporting the FV delivery to be fresh and appealing. Compared to the teachers, a lower proportion of pupils reported that the FV was fresh and appealing, with great variations in prevalence across schools (from 42% to 85% of the pupils) and even greater across classes (from 13% to 100%of the pupils). School level: By the end of the intervention, based on aggregated teacher data, a high implementation level of freshness and appearance was found at most schools (15 and 11 respectively of the 17 schools), but when aggregating the pupil data to the school level this was only found for 2 out of 20 schools. The school typologies for freshness and appearance of FV delivery showed that the majority of intervention schools (>8 schools) maintained a high supplier implementation level from the beginning to the end of the intervention period (Table 3).

Variation of the delivered FV

Individual level: On average, 87% and 64% of teachers reported that the pupils were able to taste many different fruit and vegetables. We found the same pattern for variety in fruit and vegetables in the pupil data, although the pupils’ level of prevalence was lower. The class variation was larger than the school variation. School level: Compared to the number of schools with a high implementation level of variation in vegetable delivery, twice as many schools had a high implementation level of variation in the delivered fruits (14 versus 7). School level data: Only few schools (4 concerning fruit and 1 concerning vegetables) were categorised as having high implementation levels in terms of variety according to the pupils (Table 2).

From school to class:

*Dose delivered by teachers*: Individual level: By the midway assessment, a high proportion of classroom teachers (85%) reported to offer the delivered Boost FV to their pupils *every day* but this proportion declined noticeably by the end of intervention (64%). Again we found large variations between schools. When we changed categories and looked at the proportion of teachers who offered pupils FV less than daily (*1*–*3 days a week*) close to 100% and 75% of teachers reported less than daily delivery by the two assessments (results not shown). School level: The number of schools with a high implementation level decreased (from 12 to 6 schools) from the beginning to the end of the intervention period.

From class to pupil:

*Dose received by pupils*: Individual level: Only 58% of the teachers reported that there was enough Boost FV for every pupil to get one piece every day by the midway assessment and this proportion decreased at follow-up (55%). There was a large variation between schools and between classes (0-100%). The proportion of pupils reporting an adequate amount of FV was lower than the teachers’ reports. The variation between classes was larger than between schools. Less than half of the pupils reported that they ate Boost FV every day. Data from the computer tailoring module showed that the pupils reported a high FV intake at the three measurement points (>86%). The results were confirmed when including only pupils with responses at all three assessments. In sensitivity analyses where the cut point was changed from *every day* versus *most days*/*few days or seldom*/*never to every day*/*most days*, close to 100% of the teachers reported enough FV while this was the case for almost 80% of the pupils. When the same change in cut point was made for the variable concerning pupils eating of FV, more than 80% responded this to be the case (results not displayed). School and class level: An increase in schools with high implementation levels was detected when aggregating teacher data (from 5 to 6 schools). The majority of intervention schools maintained a low implementation level (>8 schools) from the beginning to the end of the intervention period. None of the schools or the classes had a high level of dose received, measured as the frequency of pupils’ self-reported FV intake. None of the schools and only 2 classes had a high level of dose received, measured as if there was enough FV whereas this was the case at approximately one third of the schools according to the teachers.

### Implementation of the ‘Pleasant eating environment component’

Table 4 summarizes the assessment of the implementation of the ‘*Pleasant eating environment component*’.

**Table 4:** **Implementation of** ***A pleasant eating environment*** **: assessed at the individual-, school- and class level**

**Process evaluation concept**	**Source and timing of data collection**						
	**Teacher midway survey**		**Teacher follow-up survey**		**Pupil follow-up survey**		
	**Individual level average implementation level (A)**	**No of schools ≥80% implementation/out of all responding schools**	**Individual level average implementation level (A)**	**No of schools ≥80% implementation/out of all responding schools**	**Individual level average implementation level (A)**	**No of schools ≥80% implementation/out of all responding schools**	**No of classes ≥80% implementation/out of all classes**
**Fidelity/Dose delivered**							
FV are cut up in the teachers' lessons (every time/most times)	49% (0-100%)	6/19	55% (0-100%)	8/19			
Boost coordinators	47%		47%				
Other teachers	50%		58%				
How often the Boost FV were cut up					60% (A: 28-91%, B: 11-100%)	5/20	15/55
FV are usually cut up by:							
Each pupil			11% (0-100%)				
Designated FV hosts			52% (0-100%)				
Other pupils			19% (0-67%)				
Teachers			18% (0-100%)				
**Dose received**							
Time is allocated for the pupils to eat FV in class					68% (A: 40-93%, B: 24-100% )	5/20	21/55
The pupils are having a good time while eating Boost FV together			83% (0-100%)	14/20	54% (A: 35-72%, B: 21-90%)	0/20	7/55
**Reach of intervention among subgroups**							
The pupils are having a good time while eating Boost FV together							
High SEP					55%		
Medium SEP					50%		
Low SEP					63% (P = 0.0212)		
Girls					59%		
Boys					50% (P = 0.0054)		

A: School range: minimum and maximum percentage of teachers/pupils identified across schools.

B: Class range: minimum and maximum percentage of pupils identified across schools.

FV: fruit and vegetables.

SEP: socioeconomic position.

*Fidelity & dose delivered by teachers* in terms of cutting up FV during lessons was reported by only half of the teachers. The prevalence of this indicator increased slightly by the end of intervention, but there were large variation across schools. Among Boost coordinator fewer reported that they cut up FV during their lessons compared to other teachers. More frequently, the FV were cut up by pupils than by teachers. On average 60% of the pupils reported that the FV were cut up every day or most days, with large variation between schools and between classes.

*Dose received*: Individual level: On average 68 % pupils reported that time was allocated for the pupils to eat FV in class, but also here there was large between school and between class variation. On average 83% of the teachers and 54% of the pupils agreed that the pupils were having a good time when eating Boost FV together in the classroom. Again, large variations between schools and between classes were found. School level: According to the pupils, one fourth of the schools had a high implementation measured by the indicator time allocated for FV breaks. According to the teachers, 14 of the 20 schools had a high level of dose received (‘having a good time while eating FV together’) by the end of the intervention while high level of implementation at the school level was found at no schools according to the pupils’ answers.

### Reach of intervention components among various subgroups of pupils

Pupils’ daily intake of the delivered FV did not differ by SEP nor gender (Table 2). However, pupils’ experience of having a good time when eating FV in class varied significantly by SEP: More pupils from low social class had a good time when eating FV compared to high and medium social class. And more girls had a good time while eating FV in class compared to boys (Table 4).

We found no clear pattern concerning whether schools were improving and doing well for all process measures (Table 3), although some schools had stable high implementation levels or improvements for several process measures compared to other schools. No schools scored high on all process measures (Table 3).

### Characteristics of schools with a stable high level of teachers providing FV daily

Compared to schools with an increasing, decreasing or a stable low level of dose delivered, schools with a stable high level of dose delivered were more likely to 1) be a small school (83% versus 45% at the other schools); 2) have fewer pupils from families of low SEP (67% versus 27% at the other schools); 3) have a school food policy (67% versus 36% at the other schools); 4) have teachers who appreciated the intervention at both the midway evaluation and at follow-up (67% versus 36% at the other schools); 5) have pupils who appreciated the intervention (17% versus none at the other schools); 6) have a more stable FV delivery from suppliers (50% versus 44% at the other schools from September to January and 60% versus 57% from February to June); 7) have fewer teachers who cut up FV (17% versus 45%); 8) have implemented a larger number of curricular activities (67% versus 55% at the other schools) and 9) have uploaded more than half of the parental newsletters (83% versus 45% at the other schools). The differences between schools with a stable high level of dose delivered and schools with increasing, decreasing or a stable low level of dose delivered were not significant (p > 0.1618).

There was no difference between the schools with stable high level of dose delivered and the remaining schools regarding whether they had had a *FV scheme the previous school year* (17% versus 18% of the schools).

## Discussion

### This study has the following key findings:

1. The level of implementation changed during the ninth-month intervention period: *Daily provision of free FV*: The proportion of teachers experiencing a stable FV delivery increased in the first half of the intervention period and decreased in the second part of the intervention period. Freshness and appearance of FV delivered by FV suppliers improved over time. The proportion of schools in which teachers offered FV to pupils every day (dose delivered by teachers) declined, while the proportion of schools where teachers experienced that there was enough FV for all pupils every day increased over time (dose received). *A pleasant eating environment*: The proportion of teachers cutting up FV (fidelity/dose delivered) increased over time.
2. The implementation level varied greatly by schools (range for teacher measurements 0-100%, range for pupils’ measurement 28-91%) and classes (pupils’ measurements range 10-100%).
3. The daily level of intake of delivered FV did not differ by SEP or gender. More girls than boys and more pupils from low social class enjoyed the social aspects of eating FV together, compared to medium and high social class.
4. Schools with stable high teacher implementation over time in terms of daily delivery of FV to pupils were more likely to be small schools (lower number of year 7 pupils), be attended by fewer low SEP pupils; have a school food policy; have more teachers and pupils who appreciated the intervention; have a more stable FV delivery from suppliers; have fewer teachers who cut up FV; have teachers who implemented a larger number of curricular activities and uploaded more than half of the parental newsletters.

The measurements of dose delivered showed that the proportion of teachers offering FV to the pupils every day declined noticeably from the time of the midway evaluation to the end of the intervention period, when measured at individual- as well as at school level. This may reflect a declining engagement of teachers over time which to some extent may be explained by the busier schedule of teachers by the end of the school year or by teachers and pupils growing tired of the project [35]. Deteriorating implementation over time have been found in several school-based studies using educational strategies [8,14-19]. This study contributes with new knowledge in the area of environmental strategies. Still, six schools were capable of maintaining a high level of implementation during the entire implementation period. This indicates that implementation does not have to decrease over time. Reporting results exclusively at an individual level would have led to an atomistic fallacy as we would have concluded that the teacher implementation declined at all schools. This point to the importance of reporting at an individual as well as at school level to avoid atomistic as well as ecological fallacy [36].

The dose delivered by FV suppliers was low in the first two months of implementation, then reached a high level for most of the months of intervention until it declined in the last month of the intervention. In the qualitative process evaluation of the Boost FV programme, the suppliers indicated that at the beginning of the intervention, the school delivery had not yet turned into a daily routine. This was a barrier for implementation, as they simply forgot their obligation to deliver the FV [21]. This finding contributes to the understanding that an introductory period must be expected before the delivery runs efficiently. The same tendency was found for fidelity concerning the freshness and appearance of the delivered FV. This increased throughout the implementation process, and may be explained by an improvement in the communication between teachers and suppliers regarding the delivery overtime. The increase might also be due to seasonal conditions where it might have been possible to deliver fresher FV over some seasonal periods compared to others. Qualitative findings from the Boost study confirm that several schools experienced poor FV quality at the beginning of the intervention period [21]. We assume that the decline of delivery from suppliers by the end of the intervention was caused by lack of clarity concerning the end date of the delivery. However, the decrease in delivery of FV in June did not affect the outcome the follow-up data collection as this was finalised early in June in all schools and as such before any decline took place.

Our findings suggest that changes in implementation levels over time vary between the different process measures of the two intervention components. Some tend to decrease throughout the intervention period while other aspects require an introductory period before being properly implemented. These findings point to the necessity of collecting data several times during the intervention period, as well as assessing each intervention component separately to obtain a complete and accurate picture of the implementation of an intervention as highlighted by Carroll et al. (2007) [25].

From the midway survey to the follow-up study, we found a decline in dose delivered by teachers in terms of the pupils getting FV every day, but an increase in the number of teachers cutting up FV during their lessons. This may indicate that at the end of the intervention period, teachers prioritised to allocate time for cutting up FV certain days at the expense of providing pupils with a piece of FV in class on a daily basis. In this way, the two components may become barriers for the delivery of each other. This is a hypothesis to be tested in the future e.g. by interviewing teachers about the reasons for positive and negative changes in implementation levels. We also found that the average percentages for teachers’ reporting ‘*enough FV for every pupil to get one piece a day*’ was rather low compared to teachers reporting ‘*pupils offered FV 5 days a week*’. We do not know the explanation of this finding but our suggestion would be that the difference is due to a too small amount of being delivered, while delivery it timely every day.

The pupil data reflected poor implementation of the pleasant eating environment component, as large proportions of pupils (32-40%) reported that FV were not cut up every day/most days and that adequate time was not allocated for them to eat FV in class. These findings show how teachers act as gatekeepers for implementation. As the teachers have not adhered to the implementation manual (low implementation of FV break and cut up FV) there is a risk that that the intended changes in the proximal outcomes of the pleasant eating environment component such as accessibility and social norms [29] have not been achieved as intended by the project group. This should be tested in mediation analyses.

Our data suggests that schools with stable high proportions of teachers offering pupils FV every day are characterised by being smaller and having fewer pupils with few economic resources. This may well be explained by the teachers at these schools having more time and energy to engage in the FV programme. This assumption is supported by the qualitative process evaluation of the Boost FV programme in which teachers’ lack of time was identified as a main barrier for the implementation of the FV programme [21]. Likewise, other studies have identified time issues as crucial for the implementation of interventions [32,37-43]. In agreement with our study, an Australian study found that the odds for adopting a FV break were significantly higher among small schools [13]. In line with other studies [12,24,26,44-46], we found that schools with stable high levels of dose delivered were characterised by having larger proportions of pupils and teachers appreciating the Boost project.

Schools with stable high levels of dose delivered were more likely to implement the educational intervention components in terms of curricular activities and parental newsletters. This indicates that engagement in one intervention component may be linked to an overall engagement in the entire intervention. This complicates the study of a separation of effects of each intervention component of a multicomponent intervention.

Schools with a stable high level of dose delivered were more likely to have a food policy. This may reflect a greater focus on health at these schools as well as a greater level of engagement and support to the teachers’ participation in the intervention from the principals [47].

### Methodological issues

Study strengths include: a systematic and comprehensive approach to process evaluation [29], a large population of pupils, high response rates among pupils and principals, data source triangulation, teacher assessment at two different time points, assessment of implementation levels at the individual-, school-, and class-level and assessment of many process measures covering different aspects of the implementation process [9].

Study limitations include: low response rate among teachers and variations in number of teachers participating in the surveys at each school, lack of teacher data at the class level, differences in the phrasing of answer categories in questions to different data sources [48]. Furthermore the relatively small number of schools limited the power to detect significant differences in characteristics of different school types.

*Selection bias*: The low response rate among teachers challenges the generalizability of findings as it may be a certain group of teachers who have participated, probably those most engaged in the intervention. However, we do have at least one teacher response from each school and we received responses from schools reporting stable low or decreasing implementation levels, suggesting that also teachers with less engagement have completed questionnaires.

*Misclassification bias*: Responses from school principals, teachers and pupils are all at risk of *recall bias* as they have to report details that happened several months earlier. The risk is probably lower in the teacher data as several questions were asked twice during the implementation process, but they still report months back in time. *Self*-*reported* data may be affected by *social desirability bias e.g. if* teachers over report their implementation of the two components due to a feeling of obligations to the Boost project group [8,49]. This questions whether teachers are the best to rate dose delivered. Pupils’ reports’ of dose received may give a more accurate picture of the actual level of implementation [32]. Due to dichotomizing the variables used in the analyses we might have lost nuances in the respondents’ responses.

The *data source triangulation* provided us with information on the difference in experience of teachers and pupils, e.g. the appearance of the delivered FV was judged worse by pupils than teachers. A general trend for all process measures was that pupils assessed the implementation less successful compared to teachers. The differences between pupils’ and teachers’ perception of the quality and appearance of the FV delivered might be explained by the strong emphasis adolescents put on appearance and aesthetics of food [21,50-52]. Due to this emphasis the pupils may demand a greater quality of FV and the pupils may also be likely to have higher expectations concerning e.g. the variety of FV compared to the teachers. Also, the pupils are likely to have a different definition of the meaning of ‘having a good time’ in class. Another explanation may be that not exact the same wording was used in the questionnaires so they might not be fully comparable, e.g. the teachers were asked if the FV was fresh at delivery (from the FV supplier) while the pupils were asked if the FV was fresh. Pupils may have reported the freshness of FV which have been stored in class for several days while teachers may have reported the freshness at the time of delivery from suppliers.

As teachers are often responsible for several year 7 classes it was not possible to link the teacher data on implementation to specific school classes and examine between class variations in implementation levels. The aggregation of teacher information to the school level might have been too crude as pupil data showed large between class variations.

We measured pupils’ perception of implementation level only once: at follow-up. This may increase the risk of misclassification of pupil-reported implementation level. Computer tailoring was tested as a new tool for gathering process data on the pupils’ intake of the delivered FV at different times during the implementation period to supplement the information gathered at follow-up. Unfortunately, the response rate of the computer tailoring module was low and the data collection method was problematic as some pupils filled out the questionnaire in rapid succession and corrected their answers at a later point in time.

Judging from the low response rate and the risk of social desirability among teachers it seems to be an advantage to collect process measures from pupils compared to teachers.

It may be questioned whether the categorization of schools as stable low when under 80% is too high a cut point, as this cut-point leaves schools with e.g. 70% to be defined as having a stable low implementation level.

### Implication for research

Future studies should collect process data at several time points to be able to document changes in the implementation process over time. This should however be balanced with the risk of tiring the implementers with more data collections possibly leading to lower response rates.

Our collection of process data using a computer tailoring module was not successful. The response rates were low and the pupils edited their answers along the way as they were able to log on to the module as many time as they wanted to over time. However, if pupils’ are already using computer tailoring module as part of an intervention, if ways are identified to increase response rates and if pupils’ opportunities for correcting answers are eliminated, we think that the computer tailoring has a great potential as a tool to collect process measures over time.

It was the intention of the Boost study to reach boys and adolescents from low SEP at least to the same extent as other pupils [28]. The environmental component *Daily provision of free FV* was successful in reaching pupils from different social classes and boys and girls equally. It remains to be investigated if the intervention was also equally effective across gender and socio-economic background measured by the total daily FV intake (the primary outcome). Girls and pupils from low socioeconomic background appreciated a *pleasant eating environment* more than others. Other studies also find that girls emphasize the social aspect of eating together more than boys [21,52,53]. Whether the detected differences in reach of this component are linked to socially differential intervention effects will be examined in effect analyses. The effect analysis will reveal whether other approaches than a social FV break are needed to change boys’ total daily intake of FV or if it is sufficient to provide FV for free.

### Implications for practice

The increase in dose delivered by suppliers during the implementation period point to the importance of ensuring that the introduction period is long enough for implementation to take place.

As the proportion of teachers offering FV to the pupils decreased throughout the implementation period, it is important to maintain teachers’ engagement to ensure that pupils receive the intended dose. As lack of time has been found to be the main barrier for teacher implementation of a FV programme [21] one strategy to improve teachers’ engagement could be to secure the time needed for teachers to make a FV break mandatory in the national or local school curriculum.

The identification of characteristics of schools with stable high proportions of teachers providing FV to pupils every day can inform the development of strategies to increase programme implementation levels. Schools with many low SEP pupils may need more support for implementing FV programmes compared to schools with few low SEP pupils. However, we recommend that our findings on characteristics of different school types are tested in a larger school sample as our study was limited by involving 20 school units, only.

## Conclusion

The study of the implementation process of two strategies designed to increase accessibility and availability of FV at schools has the following key findings 1) we identified a large between-school and between-class variation in implementation levels, 2) the implementation levels increased for FV freshness, appearance of FV, and whether FV was cut up and declined for teachers offering pupils FV, pupils eating FV every day and pupils receiving enough FV. The FV delivery improved from baseline to midway evaluation and decreased again by the time of follow-up, 3) free FV provision reached all pupils while more girls experienced having a good time when eating FV compared to boys (gender differential reach), 4) school size, school policy, pupils’ SEP, pupils’ and teachers’ appreciation, and stable delivery of FV from suppliers seemed to be important for whether schools/teachers kept on providing pupils with FV every day during the entire intervention period. The appliance of several approaches and levels of analyses to describe data provided comprehension and knowledge of the implementation process. This is crucial for the interpretation of effect evaluations, for the development of future interventions as well as for strategies to facilitate successful implementation.
